# Chiral Amine Synthesis Using ω-Transaminases: An Amine Donor that Displaces Equilibria and Enables High-Throughput Screening**

**DOI:** 10.1002/anie.201406571

**Published:** 2014-08-19

**Authors:** Anthony P Green, Nicholas J Turner, Elaine O'Reilly

**Affiliations:** Faculty of Science and Engineering, School of Chemistry and the Environment, Division of Chemistry and Environmental Science, Manchester Metropolitan UniversityChester Street, Manchester M1 5GD (UK); School of Chemistry, Manchester Institute of Biotechnology, University of Manchester131 Princess Street, Manchester M1 7DN (UK)

**Keywords:** asymmetric catalysis, biocatalysis, chiral amines, high-throughput screening, transaminases

## Abstract

The widespread application of ω-transaminases as biocatalysts for chiral amine synthesis has been hampered by fundamental challenges, including unfavorable equilibrium positions and product inhibition. Herein, an efficient process that allows reactions to proceed in high conversion in the absence of by-product removal using only one equivalent of a diamine donor (*ortho*-xylylenediamine) is reported. This operationally simple method is compatible with the most widely used (*R*)- and (*S*)-selective ω-TAs and is particularly suitable for the conversion of substrates with unfavorable equilibrium positions (e.g., 1-indanone). Significantly, spontaneous polymerization of the isoindole by-product generates colored derivatives, providing a high-throughput screening platform to identify desired ω-TA activity.

The development of broadly applicable catalytic methods for the sustainable production of chiral amines has been identified as a key research priority by the pharmaceutical industry.[1] The importance of this challenge is highlighted by the numerous active pharmaceutical ingredients (APIs), bioactive natural products, and pharmaceutical building blocks that contain chiral amines.[2] In recent years, the development and application of enzymes as biocatalysts for the asymmetric synthesis of chiral amines has received considerable attention.[3] Examples of enzyme classes that have been utilized for their synthesis include ω-transaminases (ω-TAs),[4] ammonia lyases,[5] imine reductases,[6] amine dehydrogenases,[7] and monoamine oxidases.[8] Increasingly sophisticated techniques for enzyme evolution[9] have led to the development of engineered biocatalysts, which have been optimized for application in industrial processes.[10]

The structural simplicity and broad availability of prochiral ketones make these structures attractive starting materials for the synthesis of chiral building blocks. For example, the application of ketoreductases is currently one of the most common strategies employed for chiral alcohol synthesis.[11] The analogous asymmetric reductive amination of ketones represents a significant challenge in organic synthesis and has been highlighted as an extremely desirable transformation for use in the pharmaceutical industry.[1] ω-TAs are a family of pyridoxal-5′-phosphate (PLP)-dependent enzymes that require a sacrificial amine donor to mediate the reversible conversion of prochiral ketones into the corresponding optically pure amines.[4] The broad substrate scope and high levels of regio- and stereoselectivity associated with these enzymes make them ideal biocatalysts for chiral amine synthesis in research laboratories and in manufacturing processes. This point is highlighted by the recent application of an engineered (*R*)-selective ω-TA in a biocatalytic process for the large-scale production of the anti-diabetic API sitagliptin.[10a] Despite the enormous potential of ω-TAs, fundamental challenges associated with severe by-product inhibition and with displacing unfavorable equilibrium positions towards product formation have prevented the widespread application of these biocatalysts.[12a] The use of amine donors in large excess combined with the in situ removal of ketone by-products is generally required to achieve high yields of the desired amines (Figure 1). The most widely employed strategy uses alanine as the amine donor and relies on combinations of expensive, cofactor-dependent enzymes for pyruvate removal (Figure 1 a).[12] An elegant alternative strategy was recently described in which the ketone by-product is effectively removed by spontaneous conversion into the more stable phenol tautomer.[13] Unfortunately, the application of these approaches for scalable amine synthesis is limited by the high costs associated with their use. The preferred method for large-scale production currently involves the use of a significant excess of isopropyl amine donor in combination with the technically challenging removal of the acetone by-product by evaporation (Figure 1 bii).[10a, 14] A further challenge that complicates the development of efficient ω-TA-mediated processes is the limited availability of simple high-throughput screening methods to identify new enzymes and to evaluate large libraries of engineered variants for enhanced activity, selectivity, and stability under the required reaction conditions.[15] Herein, we describe the application of a non-chiral, low-cost amine donor that serves the dual function of efficiently displacing unfavorable reaction equilibria towards product formation whilst providing a substrate-independent high-throughput colorimetric screening method to detect desired ω-TA activity.

**Figure 1:**
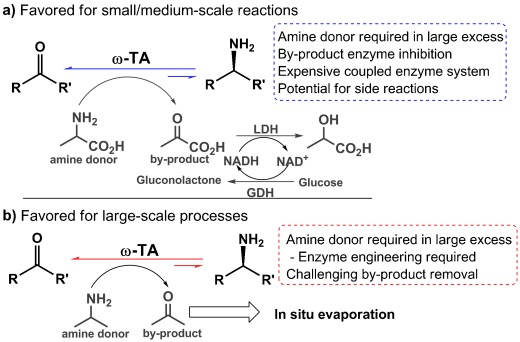
Selected examples of widely utilized conditions for small/medium-scale (a) and large-scale (b) processes that employ ω-TAs.

Commercially available *ortho*-xylylenediamine dihydrochloride (**1**) was initially evaluated as an amine donor for the biocatalytic amination of (4-fluorophenyl)acetone (**2**). Using a commercial ω-TA (ATA 113 from Codexis) as the biocatalyst, complete conversion of **2** (5 mm) into **3** (>99 %, >99 % *ee*) was achieved using one equivalent of the amine donor. Significantly, the reaction could also be carried out at a substrate concentration of 100 mm without compromising the conversion (Scheme 1). For comparison, the use of structurally related benzylamine (1.5 equiv) or L-alanine (10 equiv, no pyruvate removal) as the amine donor resulted in poor conversion (<5 %) into the desired product (Table 1). The success of the xylylenediamine donor can be attributed to the spontaneous conversion of the cyclic imine by-product **4** into the more stable aromatic isoindole **5**, effectively removing this component from the system. Transfer of an amino group from diamine **1** to the PLP cofactor to generate the required pyridoxamine-5′-phosphate (PMP) intermediate is likely to occur through rapid 5-*exo*-trig cyclization of the remaining primary amine onto an aldimine intermediate, although a stepwise hydrolysis/cyclization pathway cannot be excluded. Significantly, the isoindole by-product **5** undergoes spontaneous polymerization resulting in the formation of intensely colored derivatives,[16] providing a sensitive and operationally simple method of identifying desired transaminase activity.

**Scheme 1:**
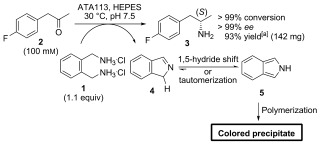
Conversion of 2 (100 mm) into (*S*)-3 using diamine donor 1 (1.1 equiv). [a] Yield of isolated product. HEPES=4-(2-hydroxyethyl)piperazine-1-ethanesulfonic acid.

**Table 1:** Conversion of 2 and 6–12 into the corresponding amines using ATA 113 and diamine 1. Comparisons to alternative donors/by-product removal systems are included. 
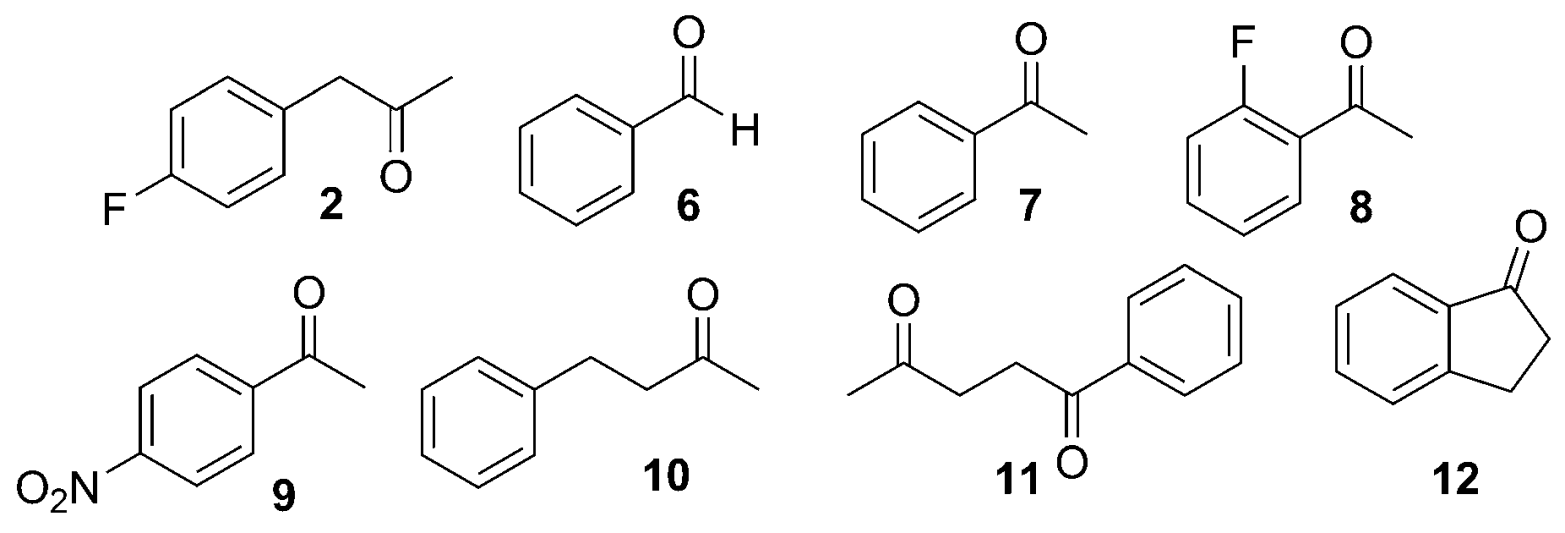

Substrate	Amine donor	Equivalents	Conv.[a]	*ee* [%]
**2**	**1**	1.0	>99	>99 (*S*)
**6**	**1**	1.1	98	n.a.
**7**	**1**	1.5	>99	>99 (*S*)
**8**	**1**	1.1	96	>99 (*S*)
**9**	**1**	1.2	97	>99 (*S*)
**10**	**1**	1.0	>99	78 (*S*)
**11**	**1**	1.0	>99	>99 (*S*)
**12**	**1**	1.5	73	>99 (*S*)
**2**	L-Ala (no p.r.)	10	<5	>99 (*S*)
**2**	Benzylamine	1.5	<5	>99 (*S*)
**12**	L-Ala (LDH/GDH)	10	21	>99 (*S*)
**12**	L-Ala (no p.r.)	100	n.d.	n.a.

[a] Conversions determined after 48 h. GDH=glucose dehydrogenase, LDH=lactate dehydrogenase, n.a.=not applicable, n.d.=not detected, p.r.=pyruvate removal system.

To demonstrate the broad utility of this method, biotransformations were carried out with benzaldehyde (**6**) and a series of prochiral ketones **7**–**12** using ATA 113 and diamine **1** (Table 1). Substrates **6**–**12** were efficiently converted into the corresponding amines with excellent conversion using <1.5 equivalents of diamine **1**. With the exception of **10**, the reactions proceeded with high selectivity (>99 % *ee*). Benzylacetone (**10**) was converted into (*S*)-(+)-1-methyl-3-phenylpropylamine with 78 % *ee* using either diamine **1** or L-alanine (10 equiv) in combination with the lactate dehydrogenase (LDH)/glucose dehydrogenase (GDH) pyruvate removal system, demonstrating that **1** does not influence the reaction enantioselectivity. Regio- and stereoselective amination of diketone **11** was followed by spontaneous cyclization into the corresponding pyrroline as described previously.[17] The efficient biocatalytic amination of 1-indanone (**12**) represents a significant challenge with existing amine donors because of highly unfavorable equilibrium positions.[12a] Impressive conversions of 54 % and 73 % into (*S*)-1-aminoindane were achieved using 1.0 and 1.5 equivalents of diamine **1**, respectively. The corresponding biotransformations using L-alanine (10 equiv) in combination with the LDH/GDH pyruvate removal system resulted in a modest 21 % conversion. In the absence of pyruvate removal, no conversion was detected using up to 100 equivalents of L-alanine.

Combined, these results clearly demonstrate the efficiency and operational simplicity of the biotransformations that use **1** as the amine donor. A further significant advantage of this system lies in the formation of intensely colored by-products, which presumably arise from spontaneous polymerization of isoindole **5** and offer a simple high-throughput screening strategy to identify desired transaminase activity. We sought to employ this assay to rapidly evaluate a panel of commercially available ω-TAs for their ability to utilize **1** as an amine donor. This panel included a series of six (*S*)-selective (Figure 2 a; L2, L4, and L6 A–F) and six (*R*)-selective (L1, L3, and L5 A–F) ω-TAs from the Codex ATA screening kit v2, which have been specifically engineered to operate using high concentrations of isopropyl amine donor and are currently the most widely utilized ω-TA biocatalysts for chiral amine synthesis. In the absence of an amine acceptor, low levels of color change were observed with a number of these biocatalysts following incubation with diamine **1** (5 mm) for three hours (L1 and L2), which is presumably due to the conversion of enzyme-bound PLP into PMP, although the presence of small quantities of ketone impurities in the commercial enzyme preparation cannot be excluded. The addition of benzylacetone (**10**; 5 mm, 1.0 equiv) as an amine acceptor resulted in significant color changes in a number of the biotransformations after only 15 minutes (L3 and L4). Following overnight incubation, reactions with all Codexis biocatalysts resulted in the formation of intensely colored solutions and significant quantities of dark precipitate (L5 and L6), demonstrating the broad utility of this amine donor for the synthesis of both the (*S*) and (*R*) enantiomers of chiral amines. In all cases, significant conversion of **10** into the corresponding amine was confirmed by GC-FID analysis (see the Supporting Information, Tables S1 and S2), demonstrating the reliability of this simple screening strategy, which provides an ideal high-throughput platform for evaluating panels of ω-TA biocatalysts for their activity towards large libraries of ketone or aldehyde substrates. In general, higher conversions were achieved with the (*S*)-selective biocatalysts than with their (*R*)-selective counterparts. Comparable levels of conversion of **10** were achieved using the corresponding immobilized ω-TA biocatalysts that were recently commercialized by Purolite/Codexis.

**Figure 2:**
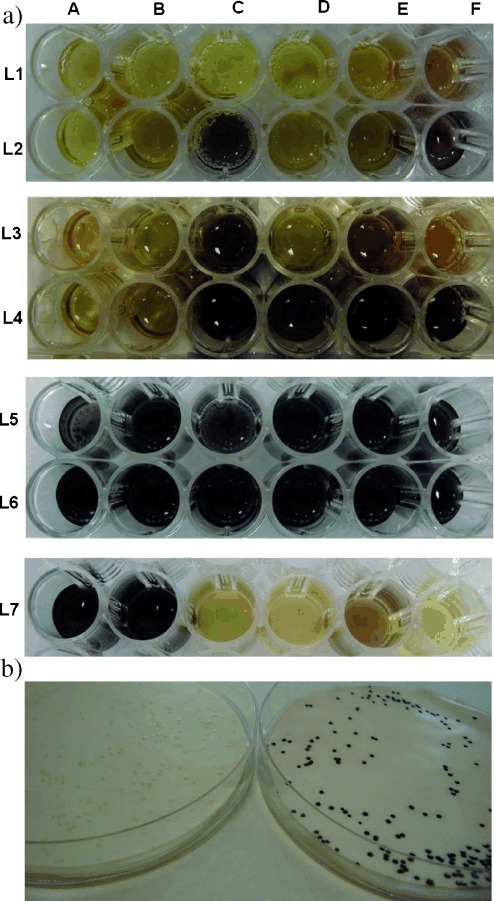
a) Conversion of 10 (5 mm) into the corresponding amine using commercially available ω-TAs and diamine 1 (5 mm). L1, L3, and L5 A–F contain the (*R*)-selective Codexis enzymes ATA 025, 303, 013, 301, 415, and 117, respectively. L2, L4, and L6 contain the (*S*)-selective Codexis enzymes ATA 254, G 05, 260, 256, 234, and 113, respectively. L1/L2: diamine 1 only, 3 h; L3/L4: 15 min after addition of substrate 10; L5/L6: 24 h after addition of substrate 10; L7: A–F=Almac TAm 106, 107, 115, 121, 125, and 140, respectively, substrate 10, diamine 1, 24 h. b) Colony-based screen with *ortho*-xylylenediamine (1). Cells expressing the *pf*-ATA gene turn dark in color after 30 min (right). Cells lacking the *pf*-ATA gene remain colorless (left).

Further evidence for the reliability of this colorimetric screening method was obtained by using commercial ω-TAs supplied by Almac (L7 A–F). Whereas the biotransformations with TAm 106/TAm 107 (L 7A and L 7B, respectively) proceeded with moderate conversion and gave intensely colored reaction mixtures, the reactions with TAm 121 (L 7D) and TAm 140 (L 7F) gave no conversion (<1 %), and the wells remained pale yellow. Significantly, low but detectable levels of color change were observed in the reactions with TAm 115 (L 7C) and TA m125 (L 7E), which were shown to proceed with <5 % and 5 % conversion, respectively (see Table S3).

Aside from providing a high-throughput method to evaluate the activity of commercial ω-TAs, the colorimetric screening strategy described above offers an ideal platform for the development of a new generation of ω-TA biocatalysts that are engineered to efficiently utilize **1** as an amine donor. Unfortunately, the protein sequence of commercial biocatalysts is rarely disclosed, necessitating the identification and application of suitable wild-type enzymes as starting points for directed evolution. We have recently reported the use of a transaminase from *Pseudogulbenkiania ferrooxidans* (*pf*-ATA) for the regio- and stereoselective amination of diketones using L-alanine as the amine donor.[17] We now demonstrate that this wild-type biocatalyst displays modest activity towards diamine **1** (see the Supporting Information). Significantly, this has allowed the development of a single-enzyme, colony-based assay to identify desired ω-TA activity. Such assays are particularly useful as they allow the rapid evaluation of large libraries of enzyme variants in a high-throughput manner. In the presence of diamine **1**, *E. coli* colonies expressing the *pf*-ATA gene rapidly became dark (Figure 2 b). Control experiments using benzylamine or diamine **1** in combination with cells lacking the *pf*-ATA gene did not lead to a color change in the colonies. The success of this assay lies in the formation of insoluble polymeric isoindole by-products, which prevent undesired diffusion. Solid-phase assays based on horseradish peroxidase (HRP)/H_2_O_2_ mediated polymerization of suitable substrates have previously been successfully exploited to engineer oxidase enzymes with enhanced biocatalytic properties.[18] The analogous assay for ω-TA activity described herein is suitable for screening genomic libraries and culture collections to identify undiscovered ω-TAs and is expected to greatly facilitate the process of engineering enzymes with high activity towards amine donor **1** and with enhanced properties for biocatalytic applications.

In summary, we have developed a simple and highly efficient process for chiral amine synthesis employing ω-TA biocatalysts. The use of *ortho*-xylylenediamine **1** serves the dual function of displacing challenging reaction equilibria towards product formation while generating intensely colored by-products, which has allowed the development of liquid-phase and colony-based assays. This method offers an ideal strategy for screening large substrate panels or libraries of ω-TA variants to facilitate the development of the next generation of ω-TA biocatalysts by directed evolution.

In memory of Phyllis O’Reilly
