# Supplementary material for: Chiral Amine Synthesis Using ω-Transaminases: An Amine Donor that Displaces Equilibria and Enables High-Throughput Screening
Source: Angew Chem Int Ed Engl. 2014 Aug 19;53(40):10714–7. doi: 10.1002/anie.201406571 (PMC4497610; doi:10.1002/anie.201406571)
Supplement: Supplementary file 1 [file anie0053-10714-sd1.pdf]

Angewandte Chemie
Eine Zeitschrift der Gesellschaft Deutscher Chemiker

Supporting Information
© Wiley-VCH 2014
69451 Weinheim, Germany

Chiral Amine Synthesis Using ω-Transaminases: An Amine Donor that Displaces Equilibria and Enables High-Throughput Screening**

Anthony P. Green, Nicholas J. Turner,* and Elaine O'Reilly**

anie_201406571_sm_miscellaneous_information.pdf

Supplementary Information

Table of Contents:

General methods and materials..S2
Analytical scale biotransformations of substrate **2** and **6-12** with ATA113 and diamine

1 or benzylamine S2

Analytical scale biotransformations of substrates **2** and **12** using ATA113 and

L-alanine in combination with the LDH/GDH pyruvate removal system...S3

Biotransformation of (4-fluorophenyl)acetone **2** with *pf*-ATA and diamine **1** S3

Preparative-scale biotransformation of (4-fluorophenyl)acetone **2** using ATA113..............................S3

^{1}H and ^{13}C NMR spectra for (*S*)-1-(4-fluorophenyl)propan-2-amine (**3**) S5

Liquid-phase screen with commercial biocatalysts...S6
Table S1...S6
Table S2 ....S7
Table S3 ....S7
Colony-based screen with *pf*-ATA S8
References...S8

General Methods and Materials

General: All biotransformations were carried out in HEPES buffer (100mM, pH 7.5) at 30 ºC. ^{1}H and ^{13}C NMR spectra were recorded on a Bruker Avance 400 spectrometer (400.1 MHz for ^{1}H and and 100.6 MHz for ^{13}C) in $CDCl_3$. The chemical shifts were recorded in ppm with the residual solvent signal referenced to 7.26 ppm and 77.23 ppm for ^{1}H and ^{13}C respectively. Coupling constants (*J*) are reported in Hz and refer to the observed peak multiplicities. GC-FID analysis was performed on an Agilent 5890 series II equipped with an autosampler and a CYCLOSIL-B (J & W scientific), 30m x 0.32mm x 0.25DF column.

Materials: Commercially available reagents were used throughout without further purification. Reagents were purchased from Sigma Aldrich, Acros or Formedium. Hybond membranes were purchased from GE Healthcare. *Escherichia coli* BL21(DE3) were purchased from Invitrogen (Carlsbad, CA). Expression vector pET-16b (69662-3) was purchased from Novagen (Darmstadt, Germany). Commercially available transaminases were kindly supplied by Almac and AstraZeneca in the form of lyophilised cell extract and as immobilized enzymes from Purolite. The transaminase gene from *Pseudogulbenkiania ferrooxidans* (*pf*-ATA) was expressed and used as previously described.[S1]

Analytical scale biotransformations of substrate 2 and 6-12 with ATA113 and diamine 1 or benzylamine

Commercially available (*S*)-selective ATA113 (1.5 mg/mL) was rehydrated in HEPES buffer (1mL, 100 mM, pH 7.5) containing PLP (2.02 mM) and xylylenediamine dihydrochloride **1** (5 mM, 5.5 mM, 6 mM, 6.5 mM or 7.5 mM). For comparison, benzylamine (7.5 mM, 1.5 equiv) and L-alanine (50 mM, 10 equiv or 500 mM, 100 equiv) were also tested as amine donors under the same reaction conditions (where necessary, the pH of the solutions were adjusted to 7.5). The substrate (5 mM from a 200 mM stock in DMSO) was added and the

mixture incubated at 30 ºC, 150 rpm in a shaking incubator. The reactions were analysed by GC-FID after 24 and 48 hours following extraction of the basified (pH 12 adjusted with 5 M NaOH) solution (100 uL) with EtOAc (1 x 300 uL) and derivatization* (acetylation).

*The reaction with substrate **11** was not acetylated prior to GC analysis.

Analytical scale biotransformations of substrates 2 and 12 using ATA113 and L-alanine in combination with the LDH/GDH pyruvate removal system

Commercially available (*S*)-selective ATA113 (1.5 mg/mL) was rehydrated in HEPES buffer (1mL, 100 mM, pH 7.5) containing PLP (2.02 mM), NAD^+ (1.5 mM), glucose (10 mg/mL, 55.5 mM), GDH (50 U), LDH (113 U) and L-alanine (50 mM, 10 equiv). The pH of the mixture was adjusted to 7.5. The substrate (5 mM from a 200 mM stock in DMSO) was added and the mixture incubated at 30 ºC, 150 rpm in a shaking incubator. The reactions were analysed by GC-FID after 24 hours following extraction of the basified (pH 12 adjusted with 5 M NaOH) solution (100 uL) with EtOAc (1 x 300 uL) and derivatization (acetylation).

Biotransformation of (4-fluorophenyl)acetone 2 with *pf*-ATA and diamine 1

pf-ATA was expressed as described previously. The 'clarified cell extract' (100 uL/mL) was added to HEPES buffer (100 mM, pH 7.5) containing PLP (2.02 mM) and xylylenediamine dihydrochloride **1** (5 mM). (**4-** Fluorophenyl)acetone (5 mM from a 200 mM stock in DMSO) was added and the mixture incubated at 30 ºC, 150 rpm in a shaking incubator. The reaction was analysed by GC-FID after 24 hours following extraction of the basified (pH 12 adjusted with 5 M NaOH) solution (100 uL) with EtOAc (1 x 300 uL) and derivatization (acetylation). The conversion after 24 hours was low, but measureable (<5%).

Preparative-scale biotransformation of (4-fluorophenyl)acetone 2 using ATA113.

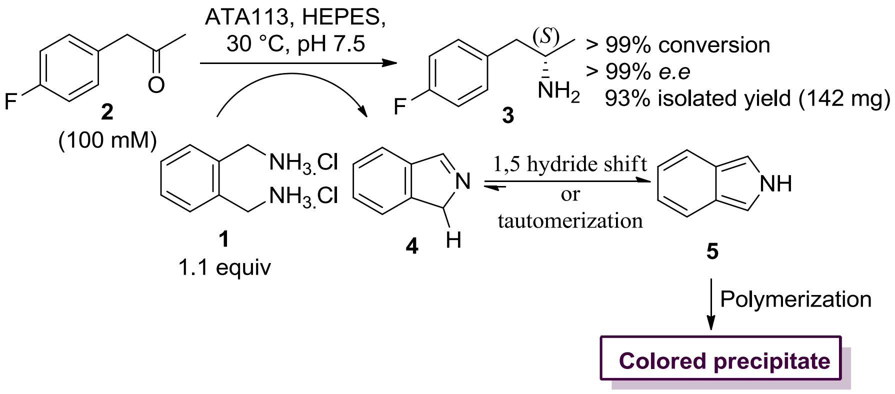

Commercially available (*S*)-selective ATA113 (5 mg/mL) was rehydrated in HEPES buffer (10mL, 100 mM, pH 7.5) containing PLP (2.02 mM) and xylylenediamine dihydrochloride **1** (230 mg, 110 mM) and the pH of the mixture was adjusted to 7.5. (4-fluorophenyl)acetone **2** (152.2 mg/133.6 uL, 100 mM, from a 1M stock in DMSO) was added and the mixture incubated at 30 ºC, 150 rpm in a shaking incubator for 24h. Having confirmed no starting material remained by GC-FID analysis (as detailed above), the solution was centrifuged at 6000 rpm for 10 minutes. The supernatant was adjusted to pH 2 and extracted with diethyl ether (1 x 30 mL) to remove any residual ketone starting material. The solution was then adjusted to pH 12 and extracted with diethyl

ether (3 x 40 mL). The organic layer was dried over $MgSO_4$, filtered and the solvent was removed *in vacuo* providing (*S*)-**3** (142mg, 93% yield, >99% *e.e.*) as a red oil which was not purified further. ^{1}H NMR (400 MHz, $CDCl_3$) δ 7.16 – 7.09 (m, 2H), 7.01 – 6.94 (m, 2H), 3.17 – 3.07 (m, 1H), 2.66 (dd, *J* = 13.4, 5.4 Hz, 1H), 2.48 (dd, *J* = 13.4, 8.0 Hz, 1H), 1.68 (br.s, 2H), 1.09 (d, *J* = 6.3 Hz, 3H); ^{13}C NMR (101 MHz, $CDCl_3$) δ 161.6 (d, *J* = 242 Hz), 135.4 (d, *J* = 3.2 Hz), 130.7 (d, *J* = 7.8 Hz), 115.3 (d, *J* = 21.0 Hz), 48.6, 45.8, 23.6.

^{1}H and ^{13}C NMR spectra for (*S*)-1-(4-fluorophenyl)propan-2-amine (3)

Biotransformation of (S)-1-(4-fluorophenyl)propan-2-amine 3 (100 mM) using ATA113

^{1}H NMR (400 MHz, $CDCl_3$) δ 7.16 – 7.09 (m, 2H), 7.01 – 6.94 (m, 2H), 3.17 – 3.07 (m, 1H), 2.66 (dd, *J* = 13.4, 5.4 Hz, 1H), 2.48 (dd, *J* = 13.4, 8.0 Hz, 1H), 1.68 (br.s, 2H), 1.09 (d, *J* = 6.3 Hz, 3H).

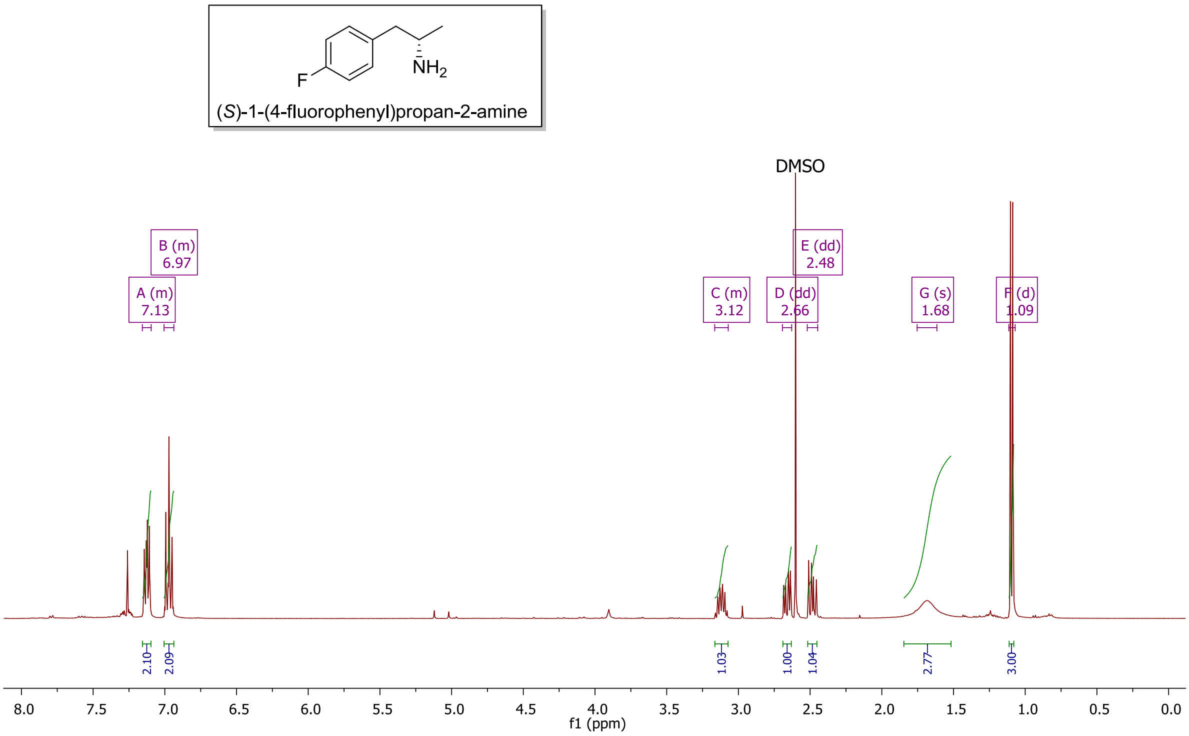

Biotransformation of (S)-1-(4-fluorophenyl)propan-2-amine 3 (100 mM) using ATA113

^{13}C NMR (101 MHz, $CDCl_3$) δ 161.6 (d, *J* = 242 Hz),135.40 (d, *J* = 3.2 Hz), 130.68 (d, *J* = 7.8 Hz), 115.26 (d, *J* = 21.0 Hz), 48.6, 45.8, 23.6.

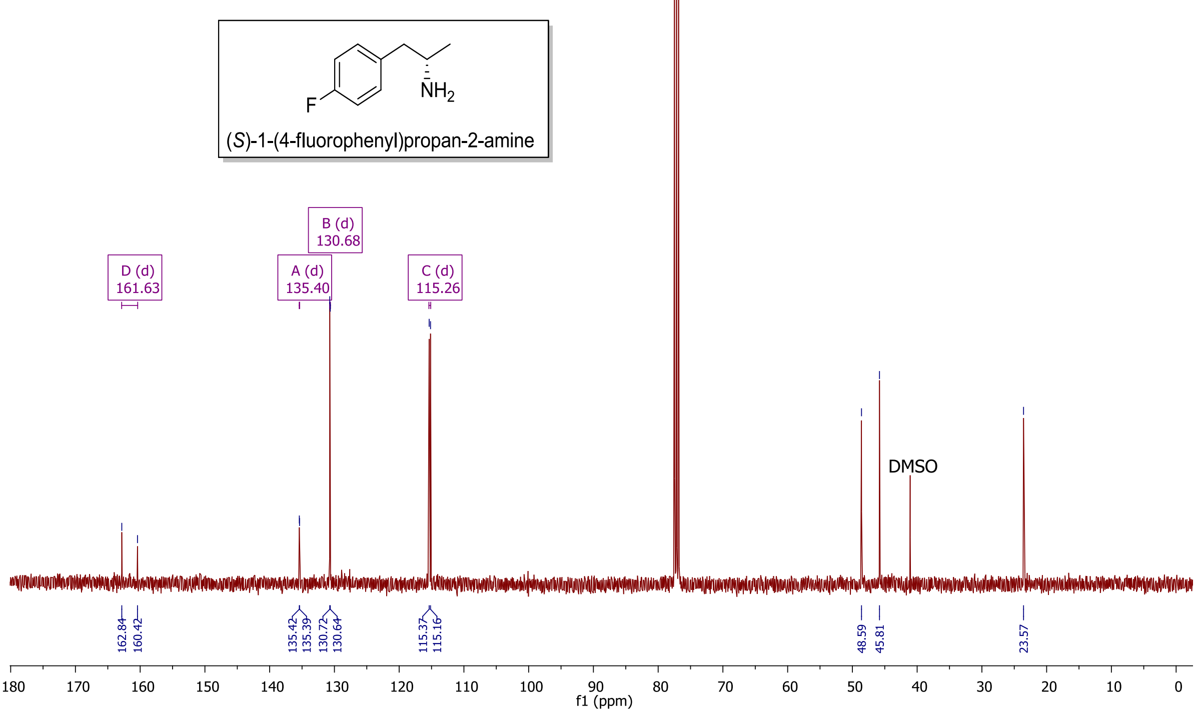

Liquid-phase screen with commercial biocatalysts

Commercially available Codexis® and Almac® ω-TAs (1.5 mg/mL) and immobilized Codexis®/Purolite® ω-TAs (40mg/mL or 8mg/mL, see manufacturers screening protocol) were rehydrated in HEPES buffer (1mL, 100 mM, pH 7.5) containing PLP (2.02 mM) and xylylenediamine dihydrochloride **1** (5 mM). The biotransformations were incubated at 30 ºC, 150 rpm in a shaking incubator for 3h. Benzylacetone **10** (5 mM) was added to the wells and the reactions were incubated for a further 24h. The reactions were monitored by color change and activity was confirmed by GC-FID after 24 and 48 hours following extraction of the basified (pH 12 adjusted with 5 M NaOH) solution (100 uL) with EtOAc (1 x 300 uL). The conversions are reported in Tables S1-3 below.

Table S1. Conversion of benzylacetone **10** to 1-methyl-3-phenylpropylamine using *o*-xylylenediamine dihydrochloride **1** and commercially available (*S*)-selective ω-transaminases

Transaminase	Biocatalyst Selectivity	Amine donor concentration	Amine acceptor concentration	Conversion
ATA254	(*S*)	5mM	5mM	98%
ATAGO5	(*S*)	5mM	5mM	>99%
ATA260	(*S*)	5mM	5mM	>99%
ATA256	(*S*)	5mM	5mM	>99%
ATA234	(*S*)	5mM	5mM	76%
ATA113	(*S*)	5mM	5mM	>99%
Purolite254	(*S*)	5mM	5mM	97%
PuroliteGO5	(*S*)	5mM	5mM	89%
Purolite260	(*S*)	5mM	5mM	99%
Purolite256	(*S*)	5mM	5mM	99%
Purolite234	(*S*)	5mM	5mM	37%

ω-TAs labelled ATA are non-immobilized Codexis® enzymes. Those labelled Purolite are the corresponding immobilized enzymes (immobilized ATA113 is not included). Only the conversions and not the enantioselectivity have been determined by GC-FID.

Table S2. Conversion of benzylacetone **10** to 1-methyl-3-phenylpropylamine using *o*-xylylenediamine dihydrocholride **1** and commercially available (*R*)-selective ω-transaminases

Transaminase	Biocatalyst Selectivity	Amine donor concentration	Amine acceptor concentration	Conversion
ATA025	(*R*)	5mM	5mM	40%
ATA303	(*R*)	5mM	5mM	65%
ATA013	(*R*)	5mM	5mM	28%
ATA301	(*R*)	5mM	5mM	21%
ATA415	(*R*)	5mM	5mM	41%
ATA117	(*R*)	5mM	5mM	26%
Purolite025	(*R*)	5mM	5mM	35%
Purolite303	(*R*)	5mM	5mM	30%
Purolite013	(*R*)	5mM	5mM	8%
Purolite301	(*R*)	5mM	5mM	20%
Purolite415	(*R*)	5mM	5mM	20%

ω-TAs labelled ATA are non-immobilized Codexis® enzymes. Those labelled Purolite are the corresponding immobilized enzymes (immobilized ATA117 is not included). Only the conversions and not the enantioselectivity have been determined by GC-FID.

Table S3. Conversion of benzylacetone **10** to 1-methyl-3-phenylpropylamine using *o*-xylylenediamine dihydrochloride **1** and commercially available Almac® ω-transaminases

Transaminase	Amine donor concentration	Amine acceptor concentration	Conversion
TAm106	5mM	5mM	18%
TAm107	5mM	5mM	35%
TAm115	5mM	5mM	<5%
TAm121	5mM	5mM	<1%
TAm125	5mM	5mM	5%
TAm140	5mM	5mM	n.d.

ω-TAs labelled TAm are enzymes which have been supplied by Almac®.
Only the conversions and not the enantioselectivity have been determined by GC-FID.
n.d. = not detected.

Colony-based screen with *pf*-ATA

A glycerol stock (made from an overnight 10mL culture in LB) containing *E. coli* BL21 (DE3) cells transformed with pET16b-*pf*-ATA was diluted 1/10,000 in sterile H_2O and plated onto a Hybond-N membrane on the surface of LB-agar supplemented with Ampicillin (100 µg/mL). Plates were incubated at 30 ºC overnight. As a control, untransformed *E. coli* BL21 (DE3) cells were grown in parallel. The recombinant protein expression was induced by adding IPTG (2 mM) to the surface of the LB-agar plates and replacing the membrane followed by incubated for a further 6 h at 30°C. Blotting paper was soaked in a solution of xylylenediamine dihydrochloride **1** (5 mM) (or benzylamine (5 mM) as a control) in potassium phosphate buffer (100 mM, pH 7.5) and the excess liquid was drained. The membranes were placed on top of the blotting paper and incubated at 30°C for 30 min.

References

[S1] E. O'Reilly, C. Iglesias, D. Ghislieri, J. Hopwood, J. L. Galman, R. C. Lloyd, N. J. Turner, *Angew. Chem. Int. Ed*. **2014**, *53*, 2447.

Complete References from Main Text

[1] D. J. C. Constable, P. J. Dunn, J. D. Hayler, G. R. Humphrey, J. L. Jr. Leazer, R. J. Linderman, K. Lorenz, J. Manley, B. A. Pearlman, A. Wells, A. Zaks, T. Y. Zhang, *Green Chem*. **2007**, *9*, 411.

[10] a) C. K. Savile, J. M. Janey, E. C. Mundorff, J. C. Moore, S. Tam, W. R. Jarvis, J. C. Colbeck, A. Krebber, F. J. Fleitz, J.Brands, P. N. Devine, G. W. Huisman, G. J. Hughes, *Science* **2010**, *329*, 305; C) T. Li, J. Liang, A. Ambrogelly, T. Brennan, G. Gloor, G. Huisman, J. Lalonde, A. Lekhal, B. Mijts, S. Muley, L. Newman, M. Tobin, G. Wong, A. Zaks, X. *J.* Zhang, *Am.Chem. Soc.* **2012**, *134*, 6467.
